# Routine versus ad hoc screening for acute stress following injury: who would benefit and what are the opportunities for prevention

**DOI:** 10.1186/1752-2897-8-5

**Published:** 2014-05-05

**Authors:** Nathaniel Bell, Boris Sobolev, Stephen Anderson, Robert Hewko, Richard K Simons

**Affiliations:** 1 Department of Surgery, University of British Columbia, 855 West 10th Avenue, Vancouver, British Columbia V5Z 1 M9, Canada; 2 School of Population and Public Health, University of British Columbia, 2206 East Mall, Vancouver, British Columbia V6T 1Z3, Canada; 3 Department of Psychiatry, University of British Columbia, 855 West 10th Avenue, Vancouver, British Columbia V5Z 1 M9, Canada; 4 College of Nursing, University of South Carolina, 1601 Greene Street, Columbia, SC 29208, USA

**Keywords:** Trauma systems, Traumatic stress/PTSD, Screening

## Abstract

**Background:**

Screening for acute stress is not part of routine trauma care owing in part to high variability of acute stress symptoms in identifying later onset of posttraumatic stress disorder (PTSD). The objective of this pilot study was to assess the sensitivity, specificity, and power to predict onset of PTSD symptoms at 1 and 4 months using a routine screening program in comparison to current ad hoc referral practice.

**Methods:**

Prospective cross-sectional observational study of a sample of hospitalized trauma patients over a four-month period from a level-I hospital in Canada. Baseline assessments of acute stress (ASD) and subsyndromal ASD (SASD) were measured using the Stanford Acute Stress Reaction Questionnaire (SASRQ). In-hospital psychiatric consultations were identified from patient discharge summaries. PTSD symptoms were measured using the PTSD Checklist-Specific (PCL-S). Post-discharge health status and health services utilization surveys were also collected.

**Results:**

Routine screening using the ASD (0.43) and SASD (0.64) diagnoses were more sensitive to PTSD symptoms at one month in comparison to ad hoc referral (0.14) and also at four months (0.17, 0.33 versus 0.17). Ad hoc referral had greater positive predictive power in identifying PTSD caseness at 1 month (0.50) in comparison to the ASD (0.46) and SASD (0.43) diagnoses and also at 4 months (0.67 versus 0.25 and 0.29).

**Conclusions:**

Ad hoc psychiatric referral process for acute stress is a more conservative approach than employing routine screening for identifying persons who are at risk of psychological morbidity following injury. Despite known limitations of available measures, routine patient screening would increase identification of trauma survivors at risk of mental health sequelae and better position trauma centers to respond to the circumstances that affect mental health during recovery.

## Background

The potential to identify trauma survivors at risk for developing PTSD and thereby enable short- and long-term mental health intervention early on was a core driving mechanism in introducing acute stress disorder (ASD) into the *Diagnostic and Statistical Manual of Mental Disorders* (DSM-IV) taxonomy in 1994 [1]. An estimated ten to forty percent of civilian injury survivors go on to display symptoms consistent with PTSD, [2-6] of which the probability of remission has been estimated at 18% to 38% [7,8]. However, the ASD diagnosis has been criticized owing to marked variability in predicting later onset of PTSD and was not recommended to be retained in the DSM-V as a predictor of subsequent PTSD [9]. While changes to the diagnosis have been made in the recent release of the DSM-V, there remains little evidence that the ASD diagnosis is accurately predicting longer-term PTSD.

These critiques have implications for how clinicians identify and manage psychological health after injury. In Canada, early screening for acute stress is not part of routine trauma care. Although it is well established that early identification of individuals at risk for PTSD is important for minimizing psychological morbidity, [10,11] trauma centers currently rely on ad hoc referral practices for identifying those individuals who display symptoms of acute stress. It is not clear whether current practices are an appropriate substitute to administering what are essentially very low-cost and easily administered self-reported surveys of acute stress. In addition, there has been a lack of literature evaluating the clinical utility of routine screening for identifying ongoing life stressors that may impact mental health. This is a significant limitation because persons with mental health illnesses often do not receive treatment, [12] are less likely to see health care specialists [13] and experience emotional disabilities often in parallel with substantial physical, social, and economic disadvantages [14,15].

We conducted a prospective observational pilot study with adult injury survivors treated at Vancouver General Hospital, British Columbia, Canada to assess whether routine screening for acute stress improved identification of patients who later went on to display PTSD symptoms compared with identification using ad hoc psychiatric referral practices currently in place. These evaluations were contextualized against patient self-reported experiences and expectations for recovery in effort to outline future surveillance and intervention opportunities for minimizing psychiatric morbidity after injury.

## Methods

This prospective cross-sectional observational pilot study targeted all residents of British Columbia and the Yukon ages 19 and older who were referred or admitted directly to Trauma Services, Vancouver General Hospital following injury. Patients were recruited over a consecutive enrollment period between June and November, 2011. Excluded were persons who had a hospital stay < 24 hours and those with a Glasgow Coma Scale < 15 at discharge. All patients actively consented to participate while in hospital. Interviews took place on the ward and on the day prior to discharge whenever possible to ensure minimal impact on patient care. Follow-up interviews were conducted 1 and 4 months after the initial interview. After each interview participants were asked if they consented to further follow-up. The follow-up period of 1 and 4 months was selected based on recommendations from the literature [16]. This study was registered and approved by the UBC Behavioral Research Ethics Board and the Vancouver Coastal Health Research Institute, which represents Vancouver General Hospital.

### Acute stress survey

Each patient completed a self-reported assessment of acute stress using the Stanford Acute Stress Reaction Questionnaire (SASRQ) [17]. The SASRQ is a 30-item closed-ended self-report questionnaire used to assess symptoms of ASD following a traumatic event [5,18,19]. The SASRQ uses DSM-IV criteria for diagnosing ASD. The SASRQ includes measures of acute dissociation (Cluster B; 10 items), re-experiencing (Cluster C; 6 items), avoidance (Cluster D; 6 items), anxiety and hyper-arousal (Cluster E; 6 items), and impairment in functioning (Cluster F; 2 items). Respondents were asked to report the frequency with which they experienced each item using a five-point Likert scale, spanning from: (0) have/did not experience, (1) very rarely experienced, (2) rarely experienced, (3) sometimes experienced, (4) often experienced, and (5) very often experienced. The diagnostic cutoff for ASD requires each person to assign a value of 3 or higher to at least three of the ten dissociative criteria, and a score of three or higher for at least one of symptom clusters C, D, E, and F. The criteria for SASD only requires meeting the diagnostic cut-off for symptom clusters C, D, and E. Positive SASD events were derived from the original SASRQ response score scores.

### PTSD survey

PTSD symptoms at one and four months were identified using the PCL-S (Specific) [20]. The PCL-S is a self-report measure developed by the US National Center for PTSD containing the 17 DSM-IV symptoms of PTSD. At each interview, participants were asked how often they have been bothered by each symptom in the past month. Responses were provided using a 5-point Likert scale, spanning from: (1) not at all, (2) a little bit, (3) moderately, (4) quite a bit, or (5) extremely. The PCL-S is scored by using a summation of the individual 17 symptoms. A cut-off score of 44 was used to identify positive PTSD symptoms. This score was chosen based on a previous sensitivity analysis using Clinician Administered PTSD Scale (CAPS) scores with 40 motor vehicle and sexual assault survivors [21].

### Behavior, service utilization, and socio-economic survey

During the baseline interview participants were asked to respond to their current employment status, level of educational attainment, living arrangement, housing tenure and if they had sought treatment for anxiety or depression in the past year. At follow-up, participants were asked if they had a post-discharge consultation with a health care provider about their health as a result of their injury, whether they were taking medication for stress, or had consulted a medical professional about anxiety or depression as a result of their injury. At the conclusion of each interview participants were asked whether they felt as emotionally or physically healthy as they were before injury, and if not, whether these experiences had been expected. All participants were asked to openly comment on these experiences as well as any barriers that they had experienced in attempting to access health care services.

### Statistical analysis

The principle outcome measures for this study were a baseline diagnosis of ASD and SASD using routine and ad hoc screening, as well the sensitivity and specificity of these practices in predicting PTSD symptomatology at 1 and 4 months. Psychiatric referrals and consultations were identified from reviewing hospital discharge summaries. Differences between means of continuous variables were examined using a 2-tailed independent samples *t* test, and differences in proportions of categorical variables were examined using a *χ*^2^ test. To account for potential type-I errors in the subgroup analysis all goodness of fit tests were reported using a continuity-adjusted *χ*^2^. All statistical analyses were generated using SAS software, Version 9.2 for Windows [22].

## Results

A total of 91 of 120 eligible patients we approached consented to participate in this pilot study, for an overall opt-in consent rate of 76%. The study participation rate at one month was 61% (n = 56) and 47% (n = 43) after four months. Six participants were lost to follow-up at month 1, but returned a survey at month 4. These response scores were included in the analyses as there was no socio-demographic, health status, or injury-related differences between those who partially or fully participated in follow-up. Socio-demographic, health status, and injury-related characteristics of all eligible patients categorized by consent, participation, and loss to follow-up status are shown in Table 1.

**Table 1:** Characteristics of the 120 patients who were eligible for enrollment by their consent to participate and by retention

**Variable**	**Consenters (n = 91)**	**Non-consenters (n = 29)**	***p value***	**Partial/full participation (n = 63)**	**Lost to follow-up (n = 28)**	***p value***
**Socio-demographics**						
Mean (SD) age (years)	45 (19)	37 (16)	0.05	45 (18)	45 (19)	0.87
Male	63 (69)	21 (84)	0.23	45 (72)	18 (62)	0.44
Having no high school diploma	11 (12)	–	–	6 (10)	5 (18)	0.45
Unememployed at time of injury	15 (16)	–	–	9 (14)	6 (21)	0.66
Currently living alone	27 (30)	–	–	13 (21)	14 (48)	0.02
Living in rented housing	61 (67)	–	–	39 (63)	22 (76)	0.32
**Health status**						
Treated for anxiety or depression in the last year	17 (19)	–	–	12 (20)	5 (17)	1.00
Scored positive for ASD	22 (24)	–	–	13 (21)	9 (31)	0.43
Scored positive for SASD	34 (37)	–	–	21 (34)	13 (45)	0.44
**Injury-related**						
Positive blood alcohol (BAC) result	13 (16)	1 (29)	0.28	8 (15)	5 (20)	0.80
Positive toxicology result	9 (11)	9 (37)	0.01	6 (11)	3 (12)	1.00
Mean (SD) injury severity score (ISS)	22 (13)	22 (10)	0.98	23 (13)	20 (12)	0.33
Intentional injury	14 (17)	4 (17)	1.00	4 (7)	10 (37)	< 0.001

Values reported are counts (% of group total) unless otherwise stated.

A total of 34 of the 91 participants (37%) scored positive for SASD and 22 participants (24%) scored positive for ASD using the SASRQ questionnaire during the baseline (in-hospital) assessment. Median hospital length of stay prior to completing the SASRQ was 7 days. All participants were screened for ASD within the time range required for an ASD diagnosis (2 – 31) days. A total of 8 of the 91 participants (9%) received a referral for a psychiatric consultation prior to discharge from Trauma Services. All eight participants had positive SASD scores and five reported positive ASD scores.

At one month, 14 of the 56 participants (25%) scored positive for PTSD symptoms based on the PCL-S assessment. At four months 12 of the 43 participants (28%) submitted positive PTSD assessment scores. Five of the 14 participants (36%) who reported positive PTSD scores at 1 month were lost to follow-up.

To assess the optimal predictive value of the ASD diagnosis we conducted a sensitivity, specificity, and power to predict test using individual ASD symptoms and different groupings for each symptom cluster. Tables 2 and 3 show the sensitivity, specificity, and power to predict PTSD symptoms at 1 and 4 months using various diagnostic groupings. Overall, employing different symptom groupings did not substantially improve either the sensitivity or predictive power of the ASD diagnosis. At month 1, the individual symptom clusters were more sensitive predictors than either the ASD or subsyndromal ASD diagnosis, but had lower predictive power. In contrast, at month 4 the individual symptom clusters were more sensitive predictors than either the ASD or subsyndromal ASD diagnosis and resulted in stronger predictive power, on average, in identifying participants who scored positive for PTSD symptoms. In both time periods, ad hoc referral practices were less sensitive than routine screening for identifying individuals who went on to display PTSD symptoms, but resulted in greater positive predictive power for identifying persons who exhibited PTSD symptoms.

**Table 2:** Sensitivity, specificity, and power to predict 9 PTSD cases at month one using the ASD symptomatology

**DSM-IV ASD criteria**	**Sensitivity**	**Specificity**	**PPV**	**NPV**
**ASD symptoms using routine screening**				
B. Dissociation	0.78	0.43	0.31	0.86
C. Re-experiencing/Intrusion	0.78	0.67	0.44	0.90
D. Avoidance	0.78	0.48	0.33	0.87
E. Anxiety/Hyperarousal	0.93	0.19	0.28	0.89
F. Impairment	0.86	0.17	0.26	0.78
**Subsyndromal ASD using routine screening**				
C + D + E	0.64	0.71	0.43	0.86
**ASD diagnosis using routine screening**				
B + C + D + E + F + 2 day	0.43	0.83	0.46	0.81
**Ad hoc referral practice**				
Consultation with psychiatry	0.14	0.95	0.50	0.77

PPV: positive predictive value; NPV: negative predictive value.

The accuracy of PTSD classification characterized by ad hoc referral with psychiatry was measured in reference to the participants SASRQ response score.

**Table 3:** Sensitivity, specificity, and power to predict 10 PTSD cases at month four using the ASD symptomatology

**DSM-IV ASD criteria**	**Sensitivity**	**Specificity**	**PPV**	**NPV**
**ASD symptoms using routine screening**				
B. Dissociation	0.75	0.39	0.32	0.80
C. Re-experiencing/Intrusion	0.50	0.58	0.32	0.75
D. Avoidance	0.58	0.55	0.33	0.77
E. Anxiety/Hyperarousal	0.92	0.13	0.29	0.80
F. Impairment	0.83	0.13	0.27	0.67
**Subsyndromal ASD using routine screening**				
C + D + E	0.33	0.68	0.29	0.72
**ASD diagnosis using routine screening**				
B + C + D + E + F + 2 day	0.17	0.81	0.25	0.71
**Ad hoc referral practice**				
Consultation with psychiatry	0.17	0.97	0.67	0.75

PPV: positive predictive value; NPV: negative predictive value.

The accuracy of PTSD classification characterized by ad hoc referral with psychiatry was measured in reference to the participants SASRQ response score.

Characteristics of participants by response scores to the PCL-S self-assessment test at month 1 and month 4 are shown in Table 4. At month 1, persons who scored positive for PTSD could be differentiated from respondents who were symptom negative when contrasted by gender (43% male vs 78% male, *p* 0.03) and by injury severity (ISS 15 vs ISS 24 *p* 0.03). No differences in health behavior, expectations, or health service utilization were observed. By month four, persons who scored positive for PTSD symptoms experienced more barriers accessing care (42% vs 10%, *p* 0.05), having unexpected physical and emotional pain (91% vs 42%; *p* 0.02), and displayed greater medication use for stress or anxiety (42% vs 6%, *p* 0.02).

**Table 4:** Characteristics of participants by response scores to the PCL-S PTSD self-assessment test at month 1 and month 4

**Variable**	**Symptom + (n = 14) month 1**	**Symptom - (n = 42) month 1**	***p value***	**Symptom + (n = 12) month 4**	**Symptom - (n = 31) month 4**	***p value***
**Socio-demographic factors**						
Mean (SD) age (years)	45 (20)	46 (19)	0.81	45 (15)	49 (20)	0.50
Male	6 (42)	33 (78)	0.03	8 (67)	22 (71)	1.00
Having no high school diploma	3 (21)	3 (7)	0.32	2 (17)	3 (10)	0.91
Unemployed at time of injury	2 (14)	6 (14)	1.00	2 (17)	4 (13)	1.00
Currently living alone	3 (21)	10 (24)	1.00	1 (8)	5 (16)	0.86
Living in rented housing	10 (71)	24 (57)	0.53	6 (50)	18 (58)	0.89
**Health status factors**						
Treated for anxiety or depression in the last year	3 (21)	8 (19)	1.00	4 (33)	3 (10)	0.17
Has consulted with GP/care provider	6 (54)	11 (38)	0.55	10 (91)	11 (42)	0.02
Currently taking medication for stress	7 (50)	15 (36)	0.53	5 (42)	3 (10)	0.05
Feel as healthy as prior to injury	3 (21)	8 (19)	1.00	4 (33)	3 (10)	0.17
Had not expected pain to still be present	12 (85)	33 (78)	0.85	9 (75)	27 (87)	0.61
Has experienced barriers obtaining care	1 (7)	2 (5)	1.00	5 (42)	2 (6)	0.02
**Injury-related factors**						
Positive blood alcohol (BAC) upon admission	1 (8)	6 (16)	0.86	2 (18)	3 (11)	0.92
Positive toxicology result upon admission	1 (8)	5 (13)	1.00	1 (9)	2 (7)	1.00
Mean (SD) Injury severity score (ISS)	15 (14)	24 (12)	0.03	18 (14)	24 (12)	0.24
Cause of injury was intentional	2 (17)	2 (5)	0.48	2 (17)	1 (3)	0.41

Values reported are counts (% of group total) unless otherwise stated.

There was an indication that participants who scored positive for PTSD symptoms at month 4 were primarily self-coping as only half of the patient population had reported discussing concerns about stress with their health care provider. When asked why, participants reported that they did not know with whom to talk with about their stress or how to approach their physician about problems they were experiencing. Differences were also observed with regards to satisfaction with current level of social support, with 33% of persons who displayed PTSD symptoms reporting they did not have adequate social support compared to 7% of persons without PTSD symptoms (*p* 0.04). When asked to comment on barriers experienced accessing care, participants listed a lack of extended health insurance and lack of income as the leading cause contributing to failure to see or consult with a health care provider about stress or anxiety.

## Discussion

Both routine screening and ad hoc referral for acute stress resulted in marked variability in predicting later onset of PTSD symptoms at 1 and 4 months after injury. It is possible that this variability may be attributed to the design of the SASRQ, the study retention and participation rate, or the opt-in as opposed to opt-out study design. The variability may also be due to a transient stress response that remitted within the first month after injury given the reduction in sensitivity, specificity, and power to predict PTSD between months 1 and 4. Although these finding suggest that the ASD diagnosis or its symptoms are not the ideal clinical detection tools for predicting later onset of PTSD or other stressors, the initial evidence suggests it is more efficient than current practice.

The sensitivity, specificity, and power to predict PTSD using the SASRQ questionnaire did produce rates that conform with the current literature. In a recent system review of thirteen adult injury-related acute stress studies, the ASD diagnosis resulted in a mean sensitivity of 0.50 (0.29 – 0.89), specificity of 0.89 (0.56 – 0.97), and power to predict PTSD of 0.54 (0.25 – 0.82) using various questionnaires over periods ranging from 2 to 24 months after injury [9]. In comparison to ad hoc clinical referral practices, routine screening improved the identification of patients whose acute stress persisted within the first month after injury. Although ad hoc referral resulted in greater predictive power after 4 months, this improvement came at the expense of identifying fewer individuals with acute stress. When contrasted against the context of patient experiences, these preliminary results suggest routine as opposed to ad hoc screening for acute stress is a more sensitive approach for identifying populations who would benefit from interventions.

Our estimation of acute stress under current practice may be conservative due to the reliance on patient discharge summary notes to identify in-hospital referrals with psychiatric services. As the level of accuracy in the number of psychiatric referrals that are recorded in patient discharge summaries is unknown, this study does provide a reference point from which to gauge future assessments. However, it may be that current practices over estimate incidence of acute stress as all persons with a previous mental health history are automatically identified in the patient registry and flagged for a consult after admission to Trauma Services. In this study, five of the eight individuals who were consulted by psychiatry prior to discharge met this criterion.

Research has shown an estimated 13 to 25% of unintentional and intentional injury survivors display symptoms of acute stress following injury [23-25]. The prevalence of acute stress identified in this study from routine screening confirms with the current literature whereas the ad hoc referral underestimates the frequency that patients experience this type of stress response. However, using the ASD diagnosis as a means to reduce the risk of PTSD remains a challenge due to limitations of screening, the lack of capacity to provide care, and the inconclusive evidence on the effectiveness of treatment [11]. In addition, it has been shown that therapies to minimize PTSD other than cognitive behavior therapy, such as behavioral activation, cognitive restructuring, counseling, relaxation therapy, stepped collaborative care, or structured writing interventions are largely ineffective [26]. While there remains a need to abstain from providing a clinical diagnosis of PTSD in absence of capacity to provide care, this reservation is not sufficient grounds to avoid routine screening for acute stress as it would detract from the capacity to understand broader contexts associated with recovery and how best to anticipate and respond to patient needs prior to and after discharge.

Our preliminary findings suggest that the definitive study should test whether an improved discharge management program improves patient outcomes. Our initial results suggest that patients lack knowledge of resources available during recovery, are dissatisfied with their level of social support to help them manage their health, under-utilizing health care services as a result of either insufficient knowledge or financial resources, and that psychological stressors are largely unexpected. Some of these experiences could be prevented through improving discharge practices. For example, in British Columbia all individuals are eligible to apply for temporary premium health care assistance to reduce the financial impact of obtaining extended health care (e.g. physiotherapy, counseling) in the event that they do not qualify for premium assistance or would exhaust their health insurance to maintain preventative health care treatment. Only one participant was knowledgeable of this insurance assistance program, suggesting that greater patient advocacy can be incorporated into routine practice. In addition, previous studies have initiated supportive self-management programs to increase the effectiveness of treatment for depression [27]. A supportive self-management program targeted at survivors who exhibit acute or long-term symptoms of stress could be tested to determine whether post-discharge care management that emphasizes coaching and behavior change can foster self-efficacy with respect to their emotional health.

We developed a systematic enrollment program to identify participants for follow-up based on daily coordination between the trauma nurse coordinator and the research team. Following rounds, the trauma nurse coordinator provided a list of all patients to be discharged in the afternoon or who were likely to be discharged on the following day. There were few occasions where more than two individuals were discharged on a single day, thereby making it feasible that one individual could conduct the initial enrollment interview. An underlining benefit of this approach is that the coordinator serves as a focal point for daily trauma operations and is in a strong position to broker additional support from clinical staff and surgical residents as to the benefits of improved patient follow-up. Limitations of this approach were that persons discharged on weekends were missed. For the definitive study, enlisting surgical residents to coordinate baseline interviews during weekends could help minimize this source of potential bias. A retrospective review of trauma registry records found no difference between consenters and patients who were missed with respect to age, sex, injury intent, BAC, or toxicology result.

Importantly, this pilot study is hindered by the limitation of a sample of 91 individuals. This limitation prohibited a more robust analysis of covariates attributed to retention and psychological morbidity, both of which could help identify whether targeted as opposed to universal screening would be more beneficial. While participants who maintained follow-up throughout the entire study period had similar health and socio-economic backgrounds, there were differences among those who withdrew from follow-up from those who maintained full or partial participation. These differences may lead to participation bias and strategies to minimize their impact will need to be addressed. It should be noted that the study was completed prior DSM-V and used a survey that was developed using the DSM-IV criteria.

## Conclusion

This pilot study suggests there are opportunities to enhance current discharge practices in trauma care by actively screening for ASD symptomatology. This would represent a practice change in trauma care, but it is no means certain other trauma centers are able or willing to take on additional screening. To our knowledge, this shift in care has not yet been undertaken elsewhere in Canadian trauma centers. Thus far, representatives of our Quality Council have encouraged continued screening for acute stress and have supported its inclusion as part of standard care practice. As such, we have proposed a quaternary discharge survey whereby acute stress assessments would become part of routine patient handover prior to discharge. The proposal was framed such that future surveys would be undertaken by our surgical residents, under the rationale that this practice emphasizes the ‘Health Advocacy’ component of the Royal College of Physicians and Surgeons of Canada CanMEDS Physician Competency Framework – traditionally one of the least supported CanMEDS roles. Further research into the epidemiology of acute stress and assessment of patient outcomes is required to assess whether this is an optimal structural framework for minimizing psychological morbidity after injury.

## Competing interests

There are no competing interests among any of the authors.

## Authors’ contributions

Conception and design: NB and RKS. Acquisition of data: NB. Analysis and interpretation of data: NB, RKS, BS, SA, RH. Drafting of manuscript: NB, RKS, BS, SA. Study supervision: RKS and BS. All authors read and approved the final manuscript.
